# Management of Gastric Varices Unsuccessfully Treated by Balloon-Occluded Retrograde Transvenous Obliteration: Long-Term Follow-Up and Outcomes

**DOI:** 10.1155/2013/498535

**Published:** 2013-12-21

**Authors:** Fumio Uchiyama, Satoru Murata, Shiro Onozawa, Ken Nakazawa, Fumie Sugihara, Daisuke Yasui, Yoshiyuki Narahara, Eiji Uchida, Yasuo Amano, Shin-ichiro Kumita

**Affiliations:** ^1^Department of Radiology, Nippon Medical School, 1-1-5 Sendagi, Center for Advanced Medical Technology, Bunkyou-ku, Tokyo 113-8603, Japan; ^2^Division of Gastroenterology, Department of Internal Medicine, Nippon Medical School, 1-1-5 Sendagi, Bunkyou-ku, Tokyo 113-8603, Japan; ^3^Department of Surgery, Nippon Medical School, 1-1-5 Sendagi, Bunkyou-ku, Tokyo 113-8603, Japan

## Abstract

Our aim was to evaluate the long-term efficacy and safety of percutaneous transhepatic obliteration (PTO) alone and combined with balloon-occluded retrograde transvenous obliteration (BRTO) for gastroesophageal varices refractory to BRTO alone. Between July 1999 and December 2010, 13 patients with gastroesophageal varices refractory to BRTO were treated with PTO (*n* = 6) or a combination of PTO and BRTO (*n* = 7). We retrospectively investigated the rates of survival, recurrence, or worsening of the varices; hepatic function before and after the procedure; and complications. The procedure achieved complete obliteration or significant reduction of the varices in all 13 patients without major complications. During follow-up, the varices had recurred in 2 patients, of which one had hepatocellular carcinoma, and the other died suddenly from variceal rebleeding 7 years after PTO. The remaining 11 patients did not experience worsening of the varices and showed significant improvements in the serum ammonia levels and prothrombin time. The mean follow-up period was 90 months, and the cumulative survival rate at 1, 3, and 5 years was 92.9%, 85.7%, and 85.7%, respectively. Both PTO and combined PTO and BRTO seem as safe and effective procedures for the treatment of gastroesophageal varices refractory to BRTO alone.

## 1. Introduction

Several modalities are currently employed for the treatment of gastric varices, including endoscopic procedures, transjugular intrahepatic portosystemic shunts (TIPS), percutaneous transhepatic obliteration (PTO), and balloon-occluded retrograde transvenous obliteration (BRTO). However, the optimal modality for the treatment of gastric varices has not yet been established [1–3].

Although endoscopic injection of n-butyl-2-cyanoacrylate (NBCA), a tissue adhesive agent, is an effective first-line treatment for bleeding gastric varices [4, 5], this method carries a potential risk of migration of NBCA from the varices to the systemic venous circulation, especially in patients with fundal varices associated with a large gastrosystemic venous shunt. This migration may result in fatal complications such as pulmonary embolism [6]. TIPS placement is the second-line treatment for gastroesophageal varices, and it is effective in reducing portal pressure. TIPS has been widely used in patients with variceal bleeding or refractory ascites associated with portal hypertension [7]. However, it has a major drawback, that is, the development of postplacement hepatic encephalopathy, which reportedly occurs in approximately 20% of patients [7]. Therefore, the clinical effectiveness of TIPS placement for gastric varices is controversial.

The BRTO procedure was introduced for the obliteration of portal-systemic venous shunts, and it has become widely accepted as a minimally invasive, highly effective treatment for gastric varices [1, 8]. However, BRTO is not suitable for gastric varices that lack a main draining vein, as they cannot be catheterized. Moreover, gastric varices that have a direct connection with the coronary vein and esophageal varices are also difficult to treat using BRTO. In these cases, PTO or a combination of PTO and BRTO may be useful.

Thus, the purpose of this study was to evaluate the efficacy and safety of PTO or a combination of PTO and BRTO for gastric varices that could not be treated by BRTO alone.

## 2. Materials and Methods

### 2.1. Patients

Between July 1999 and December 2010, 76 patients with gastric varices and uncontrolled hepatic encephalopathy or uncontrolled gastric varices despite endoscopic treatment were referred to our department to undergo BRTO. Of these, 13 (17%; 8 men and 5 women; age range: 36–79 years; mean age: 62 years) could not be successfully treated by BRTO alone. From among these 13 patients, 6 did not have a well-developed gastrorenal shunt suitable for catheterization. The remaining 7 patients had a well-developed gastrorenal shunt, but their varices had other large outflow shunt vessels and included esophageal varices, which BRTO would not be able to sclerose sufficiently. Endoscopic findings showed tortuous, winding varices (F1) in 2 patients, nodular-shaped varices (F2) in 1 patient, and large, tumorous varices (F3) in 10 patients; red spots were observed in 5 patients, and uncontrolled hepatic encephalopathy with extraportosystemic shunts was observed in the 2 patients with F1. Patient characteristics are shown in Table 1.

All patients provided informed consent prior to undergoing treatment. This study was conducted retrospectively in accordance with the ethics guidelines of the Declaration of Helsinki and the International Conference on Harmonization Guidelines for Good Clinical Practice.

### 2.2. Treatment Strategy for Gastric Varices

As recommended by the treatment algorithm, all patients first underwent BRTO. When this procedure could not obliterate the gastric varices because of collateral venous drainage, PTO was selected as the second line of treatment and conducted on another day. When PTO was not successful in alleviating the gastric varices, the patients were treated with a combination of PTO and BRTO as the third line of treatment.

### 2.3. PTO Technique

**Figure 1:**
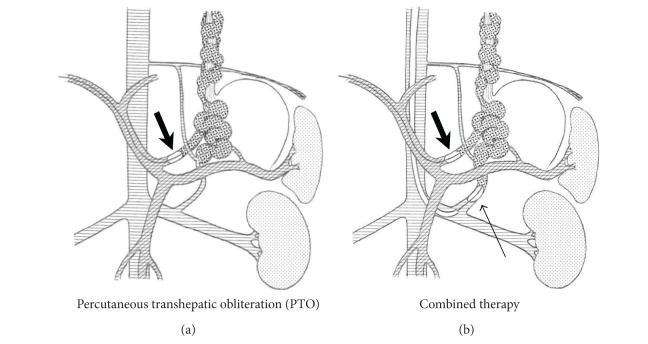
Schemas of percutaneous transhepatic obliteration and combined therapy. Percutaneous transhepatic obliteration (PTO): in the case of varices whose gastrorenal shunts were too underdeveloped for balloon occlusion, the PTO procedure was used. The intrahepatic portal vein is punctured from the right or left upper abdomen using a PTCD needle under ultrasonographic guidance. After an introducer sheath is inserted into the intrahepatic portal vein, a balloon catheter is inserted, and the catheter is advanced into the inflowing shunt vessel (mainly the left gastric vein), after which the balloon was inflated to occlude blood inflow (broad arrow). Combined therapy: the combined PTO and balloon-occluded retrograde transvenous obliteration (BRTO) procedure was used when the gastric varices had large shunt vessels (usually gastrorenal and left gastric vein shunts), which could not be successfully treated with either treatment alone. The narrow arrow indicates a balloon catheter placed in the gastro-renal shunt.

Before PTO, ultrasonography was performed for all patients in order to determine the best access route into the portal venous system. Percutaneous transhepatic puncture of the intrahepatic branch of the portal vein was performed using an 18-gauge needle under sonographic guidance after local anesthesia, and a 5- or 8-French sheath catheter was introduced into the portal vein. Direct portography was performed to identify the feeding and draining veins of the gastric varices. The gastric varices often had multiple feeding veins, and a coaxial catheter was inserted into these feeding veins while avoiding the main feeding vein. The feeding veins were embolized with microcoils or an emulsion of Lipiodol (Guerbet, Paris, France) and NBCA (Histoacryl; Braun, Melsungen, Germany). Subsequently, a 5-French balloon catheter with a diameter of 9 mm and effective length of 70 cm (Serecon MP catheter II; Terumo-Clinical, Tokyo, Japan) or an 6-French balloon catheter with a diameter of 20 mm and effective length of 70 cm (Serecon MP catheter II; Terumo-Clinical) was advanced into the main feeding vein, and venography was performed with the balloon inflated. When the gastric varices were visualized and their retention of the contrast medium was confirmed, 5% ethanolamine oleate solution mixed with iopamidol (EOI) was slowly injected into the gastric varices in the antegrade direction under fluoroscopic guidance. Immediately prior to the treatment, 4000 U of haptoglobin (Yoshitomi, Osaka, Japan) was administered by drop infusion to protect against potential renal damage from the EOI sclerosant [11]. When the varices could be visualized in their entirety, EOI injection was stopped. Portography by hand injection was repeated to assess obliteration of the feeding veins and to confirm the absence of flow to the gastric varices 4 hours after the injection of EOI (Figure 1).

### 2.4. Combined PTO and BRTO

**Figure 2:**
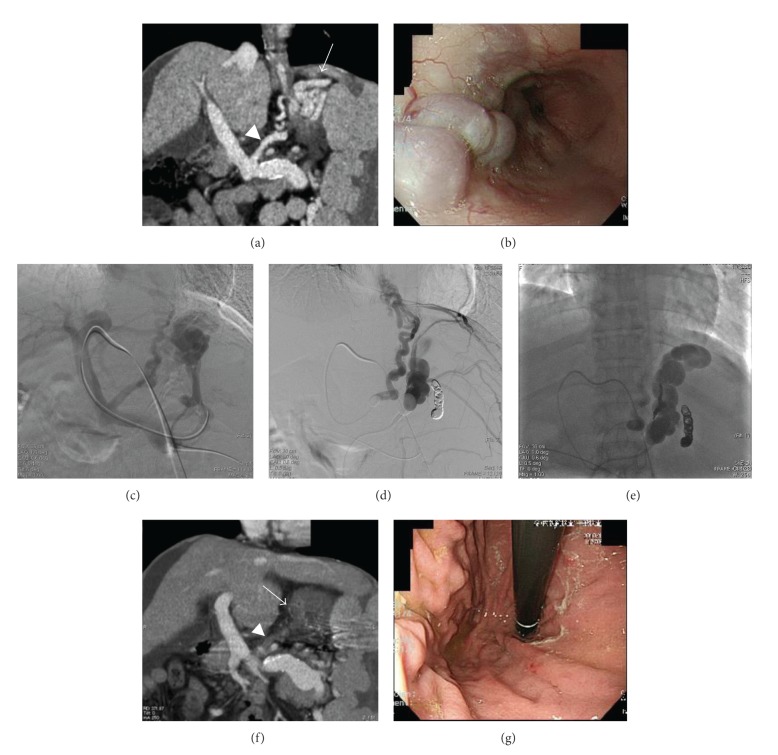
A 36-year-old woman with gastroesophageal varices. The patient visited our outpatient clinic because of general fatigue. Hematologic tests showed liver dysfunction and pancytopenia, and the gastroesophageal varices tended to worsen despite several sessions of endoscopic therapy. An enhanced computed tomography (CT) scan coronal image (a) showed dilated and tortuous veins at the gastric fundus (arrow), and large gastric varices supplied by the left gastric vein (arrowhead). Endoscopic examination of the stomach (b) showed significant large tumorous gastroesophageal varices (F3). Such varices pose a high risk of variceal bleeding and are an indication for embolotherapy. This patient underwent combined balloon-occluded retrograde transvenous obliteration (BRTO) and PTO therapy. Direct portography (c) showed gastroesophageal varices from the left gastric vein and posterior gastric vein. Balloon-occluded left gastric venography and left adrenal venography after embolization of the posterior gastric vein with platinum coils showed the left inferior phrenic vein and intercostal vein as collateral vessels (d). Fluoroscopic image obtained at 4 hours after embolotherapy shows the varices filled with iopamidol (e). An enhanced coronal CT scan obtained 14 days after embolotherapy showing the gastric varices and the left gastric vein as a low-density area ((f), arrow and arrowhead), suggesting complete obliteration. Endoscopic examination conducted 6 months after the embolotherapy showed eradication of the gastroesophageal varices (g).

A combined PTO and BRTO procedure was used when the gastric varices had large shunt vessels (usually gastrorenal and left gastric vein shunts), which could not be successfully treated using either procedure alone. After administration of 4000 U of haptoglobin, 5% EOI was slowly injected into the gastric varices in the antegrade direction under fluoroscopic guidance. EOI was administered to both the gastrorenal shunt and the left gastric vein through the occluded balloons. Destruction of the feeding veins and absence of flow to the gastric varices was again assessed using portography by hand injection at 4 hours after the injection of EOI (Figures 1 and 2).

### 2.5. Evaluation of Procedure Efficacy and Follow-Up

In the immediate evaluation, nonvisualized varices were examined by direct portography after embolotherapy. Follow-up evaluation included assessment of recurrence and bleeding of gastric varices, worsening of esophageal varices, complications, and the rate of survival. In addition, hepatic function tests including those for serum albumin, bilirubin, and ammonia concentrations and determination of prothrombin time 7–10 days after embolotherapy as well as tests for the presence of symptoms were conducted during the follow-up period. Changes in the Child-Pugh score before and after the procedure were also evaluated. The varices were examined by endoscopy and/or computed tomography (CT) in the portal phase before and 7–14 days after the interventional embolotherapy. Endoscopic findings for varices were evaluated by endoscopists according to the general rules proposed by the Japanese Research Society for Portal Hypertension [12].

The follow-up period for the recurrence of gastroesophageal varices was measured in days from the date of the procedure until the first date when endoscopy showed morphologic recurrence of gastroesophageal varices or the date of the most recent endoscopic examination. Similarly, the follow-up period for bleeding of gastric varices was calculated in days from the date of the procedure until the date when bleeding occurred or the date of the most recent clinical visit without a bleeding episode. Lastly, the survival period was measured in days from the date of the procedure until the date of death or the most recent clinical visit.

### 2.6. Statistical Analysis

The cumulative survival rate and nontreatment rate of gastric varices were calculated using the Kaplan-Meier method, and the values of hepatic function tests before and after the procedure were compared using the paired *t*-test. All statistical testing was performed using SPSS version 18 software (SPSS Japan, Inc., Tokyo, Japan), and *P* < 0.05 was considered statistically significant.

## 3. Results

### 3.1. Evaluation of the Procedure and Follow-Up

Seven sessions of combined PTO and BRTO therapy were performed for 7 patients (1 session each), and 7 sessions of PTO therapy were performed for 6 patients. Direct splenoportography or venography after embolotherapy showed complete obliteration of the gastric varices in all 13 patients (Table 2). At the follow-up 3–6 months after embolotherapy, enhanced CT and endoscopic examination showed that the gastroesophageal varices were completely absent in 7 patients (4 underwent PTO and 3 underwent combined PTO and BRTO), while the severity of the submucosal varices was significantly reduced in 6 patients (2 underwent PTO and 4 underwent combined PTO and BRTO). Additionally, 2 of these 6 patients received subsequent endoscopic injection sclerotherapy (EIS) for small, residual esophageal varices. Endoscopic findings showed F0 in 7 patients, F1 in 5 patients, and F2 in 1 patient, with complete absence of red spots.

During the follow-up period, the gastroesophageal varices recurred in 2 patients. Of these, 1 with hepatocellular carcinoma (HCC) experienced recurrence because of a portal venous tumor thrombus 11 months after the initial PTO therapy. Therefore, she underwent a second PTO procedure. However, she died 15 months after the initial PTO therapy because of HCC progression. The second patient experienced rebleeding 7 years after combined PTO and BRTO therapy. This patient had visited our outpatient clinic for the first 5 years after treatment but was lost to follow-up in the next 2 years and died suddenly because of variceal rebleeding. The remaining 11 patients did not experience worsening of the gastroesophageal varices and did not undergo further embolotherapy during the follow-up period of 5–167 months (mean, 90 months). The nontreatment rate at 1, 3, 5, and 7 years was 100%, 91.7%, 91.7%, and 81.5%, respectively.

### 3.2. Changes in Hepatic Function

None of the patients enrolled in this study showed a decline in hepatic function following treatment (Table 3). Overall, the serum ammonia levels improved significantly (*P* = 0.0061, paired *t*-test) from 107.5 *μ*g/dL (range, 27–276) to 47.3 *μ*g/dL (23–66). Similarly, the prothrombin time also significantly improved (*P* = 0.032, paired *t*-test) from 58.2% (range, 42.1%–87.1%) to 66.9% (52.5%–88.4%). All other measured parameters remained unchanged, and the hepatic encephalopathy subsided in all 5 previously affected patients. A nonsignificant change (*P* = 0.109, Wilcoxon *t*-test) in the Child-Pugh score was observed after the procedure (before: A/B/C = 3/7/3; after: A/B/C = 4/9/0).

### 3.3. Therapy-Related Complications

No major procedure-related complications such as bleeding were observed. The main complications after treatment were fever (*n* = 11) and abdominal pain (*n* = 7). A transient fever with body temperature higher than 38°C was observed in 3 patients, and abdominal pain requiring medicinal therapy was observed in 2 patients. All these patients were treated conventionally, and both the fever and abdominal pain subsided within 1 week. Hemoglobinuria was also observed in 3 patients, but this symptom subsided within 2 days. Newly developed ascites or aggravated ascites were observed on CT in 2 patients, and newly developed pleural effusion was observed on CT in 1 patient.

### 3.4. Survival Rates

During the follow-up period, 3 patients died: 1 died because of liver failure 5 months after PTO therapy, although the gastroesophageal varices did not worsen in this patient, another patient died because of progression of HCC 15 months after the initial PTO therapy, and the last patient died because of rebleeding 7 years after PTO therapy. The mean follow-up period was 90 months (5–167 months), and the cumulative survival rate at 1, 3, 5, and 7 years was 92.9%, 85.7%, 85.7%, and 77.1%, respectively.

## 4. Discussion

Although gastric varices generally have a lower bleeding rate than esophageal varices [3, 13], once ruptured, gastric varices tend to bleed profusely. Further, while the mortality rate associated with esophageal variceal hemorrhage is 6%–15% [14, 15], it increases significantly to 45–55% in the case of gastric variceal hemorrhage [9]. Therefore, gastric varices are potentially life threatening for patients with liver cirrhosis.

BRTO was developed in Japan, and it has been widely adopted in the country [1, 12, 16]. It is an interventional technique for variceal sclerotherapy of gastric varices that are difficult to treat endoscopically. BRTO has been applied in the treatment of gastric varices with a high rate of technical success (87%–100%) and a low incidence of major complications [2, 17]. The 5-year survival rate for BRTO is reported to be 85% [18], whereas that for TIPS has been reported to be 51% [19]. However, some gastric varices that lack a catheterizable main draining vein or those that have a direct connection with the coronary vein and esophageal varices are difficult to treat with the BRTO procedure.

Reports of refractory varices treated with BRTO are rare [2, 17], and an adequate treatment strategy for such varices has not yet been established. In the present study, we performed PTO or a combination of PTO and BRTO in cases of refractory varices. A recent study suggested that the technical success rate and postprocedural bleeding rate of PTO are comparable with those of BRTO [20]. Moreover, combined PTO and BRTO therapy is similar to PTO or BRTO in terms of the therapeutic effects and short-term prognosis [20]. However, long-term follow-up studies comparing therapeutic efficacy, complication rates, recurrence of gastroesophageal varices, changes in liver function and hepatic encephalopathy, and cumulative survival rates are limited.

Although the study population in the present study was small, the therapeutic efficacy was 100%, recurrence rate was 15% (17% for PTO and 14% for combined PTO and BRTO), and the cumulative survival rates at 1, 3, and 5 years were 92.9%, 85.7%, and 85.7%, respectively. In contrast to the present study, previous studies have found that BRTO alone is associated with a therapeutic efficacy of 87%–100%, a recurrence rate or rate of variceal aggravation of 24.9%–58% [2, 18], and cumulative survival rates at 1, 3, and 5 years of 100%, 100%, and 85%, respectively [18]. Further, the recently reported statistics for TIPS are as follows: therapeutic efficacy, 50–63%; rate of recurrence or variceal aggravation, 61–69%; cumulative survival rates at 1, 2, and 4 years, 75%, 69%, and 60%, respectively [21].

Generally, obliteration of outflow from the portal vein may exacerbate portal hypertension and aggravate ascites and esophageal varices. In this study, the hepatic encephalopathy and prothrombin time improved after combined PTO and BRTO therapy and liver function did not differ significantly before and after the procedures. Further, Child-Pugh C patients (*n* = 3) showed improved scores after either PTO or combined PTO and BRTO therapy. However, fever and abdominal pain were commonly, although transiently, observed in the present study at relatively high rate. Occurrence or exacerbation of pleural effusion and ascites with portal hypertension was observed, but these conditions were controllable by medical treatment. Unlike TIPS, BRTO increases hepatopetal blood flow by obliteration of the hepatofugal shunt vessels, and it may subsequently improve the blood flow in the liver parenchyma [22, 23]. Therefore, it may aggravate portal hypertension but improve liver function biochemically [24]. We expected that PTO and the combination of PTO and BRTO would also obliterate hepatofugal shunts and produce similar changes in hemodynamics. EIS after embolotherapy aids in the management of recurrent or aggravated varices, because esophageal varices are generally more controllable with endoscopic therapy than gastric varices are [3, 9, 14, 15].

The limitations of our study include its retrospective nature, the limited numbers of patients, and the absence of evaluation of portal pressure changes or blood flow changes before and after the procedures. Moreover, 2 patients received subsequent EIS for small, residual esophageal varices.

## 5. Conclusion

Both PTO and combined PTO and BRTO therapy appear to be safe and effective for the treatment of gastric varices that cannot be managed by BRTO alone.

## Figures and Tables

**Table 1:** Patient characteristics.

	Patients (*n* = 13)
Age (years)	62 (36–79)
Sex (male/female)	9/4
Etiology of cirrhosis	
HBV/HCV/both	1/9/0
Alcohol	3
Presence of hepatocellular carcinoma	1
Child-Pugh classification	
A/B/C	3/7/3
Location of gastric varices*	
Isolated gastric varices	5
Gastroesophageal varices	8
Form of gastric varices^#^	
F1/F2/F3	2/1/10
Presence of red spots	5
Encephalopathy	5
Gastrorenal shunt	7
History of variceal bleeding	4
Follow-up period (mean)	90 months (5–167)

HBV: hepatitis B virus; HCV: hepatitis C virus.

*Locations of gastric varices were based on the criteria proposed by Sarin et al. [9].

^#^Forms of gastric varices were graded by the classification described by Hashizume et al. [10].

F1: tortuous, winding varices; F2: nodular varices; F3: large tumorous varices.

**Table 2:** Results of the 13 patients treated with PTO or Combined PTO and BRTO.

	PTO (*n* = 6)	com-T* (*n* = 7)	Total (*n* = 13)
Complete disappearance			
After embolization	6	7	13 (100%)
Follow-up (3–6 months)	4	3	7 (54%)
Significant reduction			
Follow-up (3–6 months)	2	4	6 (46%)
EIS** after embolotherapy	0	2	2 (15%)
Form of gastric varices (F0/F1/F2/F3)			
Before	0/1/0/5	0/1/1/5	0/2/1/10
After	3/2/1/0	4/3/0/0	7/5/1/0
Recurrence of gastroesophageal varices	1	1	2 (15%)
Postprocedural bleeding	0	1	1 (8%)
Death	2	1	3 (23%)
Cause of death			
Hepatic failure	1	0	1 (8%)
Hepatocellular carcinoma	1	0	1 (8%)
Bleeding of esophageal varices	0	1	1 (8%)

*Combined PTO and BRTO.

**Endoscopic injection sclerotherapy.

**Table 3:** Changes in hepatic functions.

	PTO		P*	Combined PTO and BRTO		P*
	Before	After		Before	After	
Hepatic function						
Serum albumin (g/dL)	2.6–3.9 (3.4)	2.8–4.0 (3.3)	0.0873	2.2–3.3 (3.1)	2.8–3.7 (3.1)	0.481
Total bilirubin (mg/dL)	1.0–3.8 (1.8)	0.8–2.6 (1.7)	0.391	0.6–3.1 (1.4)	0.6–2.8 (1.2)	0.238
Prothrombin time (%)	47.1–87.1 (62.5)	52.5–85.7 (62.8)	0.472	42.1–71.4 (53.8)	57.8–88.4 (71.0)	0.0162
Ammonia (*µ*g/dL)	27–276 (127.5)	23–66 (45.8)	0.0459	56–121 (87.5)	33–63 (48.8)	0.00154
Encephalopathy	4	0	—	1	0	—
Child-Pugh classification						
A/B/C	2/2/2	2/4/0	0.159**	1/5/1	2/5/0	0.207**

*Between before and after PTO, hepatic function data were analyzed using the paired *t*-test.

**Between before and after PTO, Child-Pugh classification was analyzed using the Wilcoxon *t*-test.
